# Supplementary material for: Branched-chain amino acids modulate the proteomic profile of Trypanosoma cruzi metacyclogenesis induced by proline
Source: PLoS Negl Trop Dis. 2024 Oct 9;18(10):e0012588. doi: 10.1371/journal.pntd.0012588 (PMC11493278; doi:10.1371/journal.pntd.0012588)
Supplement: S1 Fig — Metacyclogenesis efficiency of parasites differentiated in TAU 3AAG, Pro, Pro-Leu, Pro-Ile and Pro-Val. Graph shows average and standard deviation of three biological replicates. Statistically analysis using differentiation rate in TAU Pro as control was performed applying one-way ANOVA with Turkey’s multiple comparisons test (a = 0.05). (PDF) [file pntd.0012588.s001.pdf]

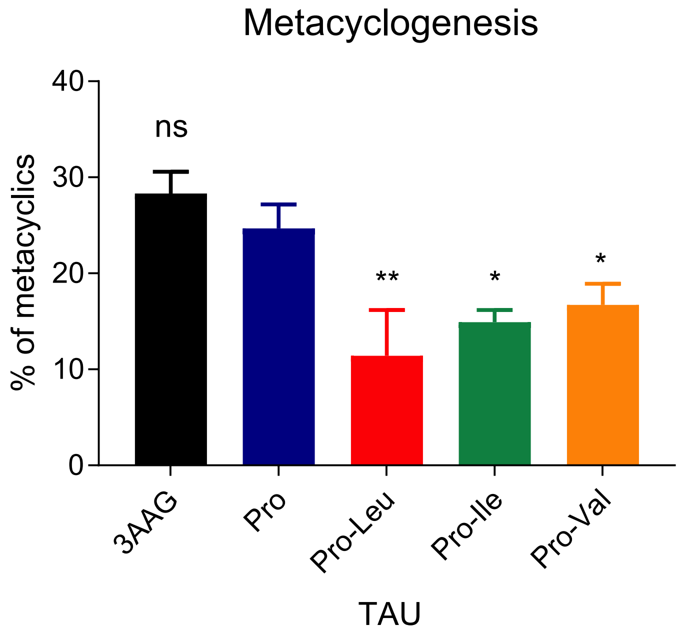

Figure S1: Presence of BCAAs affect metacyclogenesis induced in TAU Pro. Metacyclogenesis efficiency of parasites differentiated in TAU 3AAG, Pro, Pro-Leu, Pro-Ile and Pro-Val. Graph shows average and standard deviation of three biological replicates. Statistically analysis using differentiation rate in TAU Pro as control was performed applying one-way ANOVA with Turkey's multiple comparisons test (a=0.05).
