# Supplementary material for: Branched-chain amino acids modulate the proteomic profile of Trypanosoma cruzi metacyclogenesis induced by proline
Source: PLoS Negl Trop Dis. 2024 Oct 9;18(10):e0012588. doi: 10.1371/journal.pntd.0012588 (PMC11493278; doi:10.1371/journal.pntd.0012588)
Supplement: S2 Fig — Volcano plot of LFQ values of proteins from metacyclic trypomastigotes differentiated in TAU Pro and TAU 3AAG. Differentially expressed proteins are shown in orange (down-regulated in TAU Pro) and green dots (up-regulated in TAU Pro). Pie charts show the number of hypothetical proteins in the sets of up and down-regulated proteins. (PDF) [file pntd.0012588.s002.pdf]

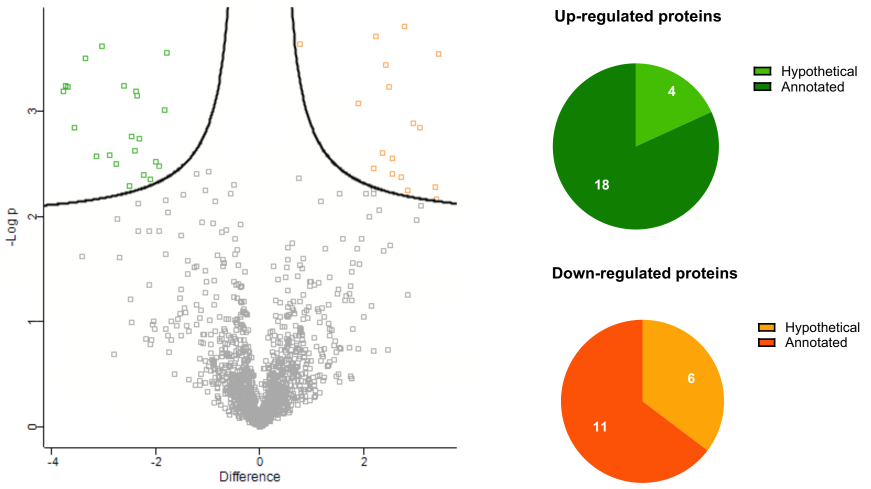

Figure S2: TAU-3AAG and TAU-Pro induce similar proteomic profiles in metacyclic trypomastigotes. Volcano plot of LFQ values of proteins from metacyclic trypomastigotes differentiated in TAU Pro and TAU 3AAG. Differentially expressed proteins are shown in orange (down-regulated in TAU Pro) and green dots (up-regulated in TAU Pro). Pie charts show the number of hypothetical proteins in the sets of up and down-regulated proteins.
