# Supplementary material for: Branched-chain amino acids modulate the proteomic profile of Trypanosoma cruzi metacyclogenesis induced by proline
Source: PLoS Negl Trop Dis. 2024 Oct 9;18(10):e0012588. doi: 10.1371/journal.pntd.0012588 (PMC11493278; doi:10.1371/journal.pntd.0012588)
Supplement: S3 Fig — The frequency of each GO was normalized over the number of differentially expressed proteins in each group (Pro-Leu, Pro-Ile and Pro-Val). (PDF) [file pntd.0012588.s003.pdf]

A

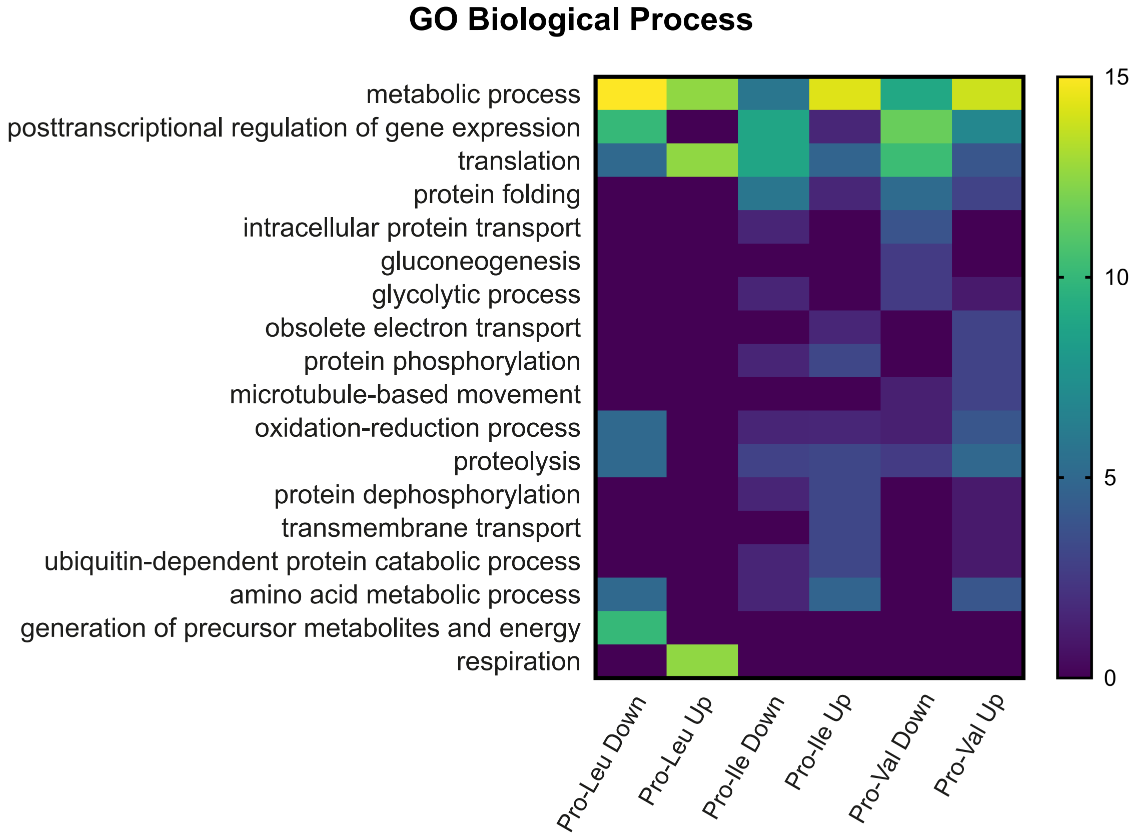

B

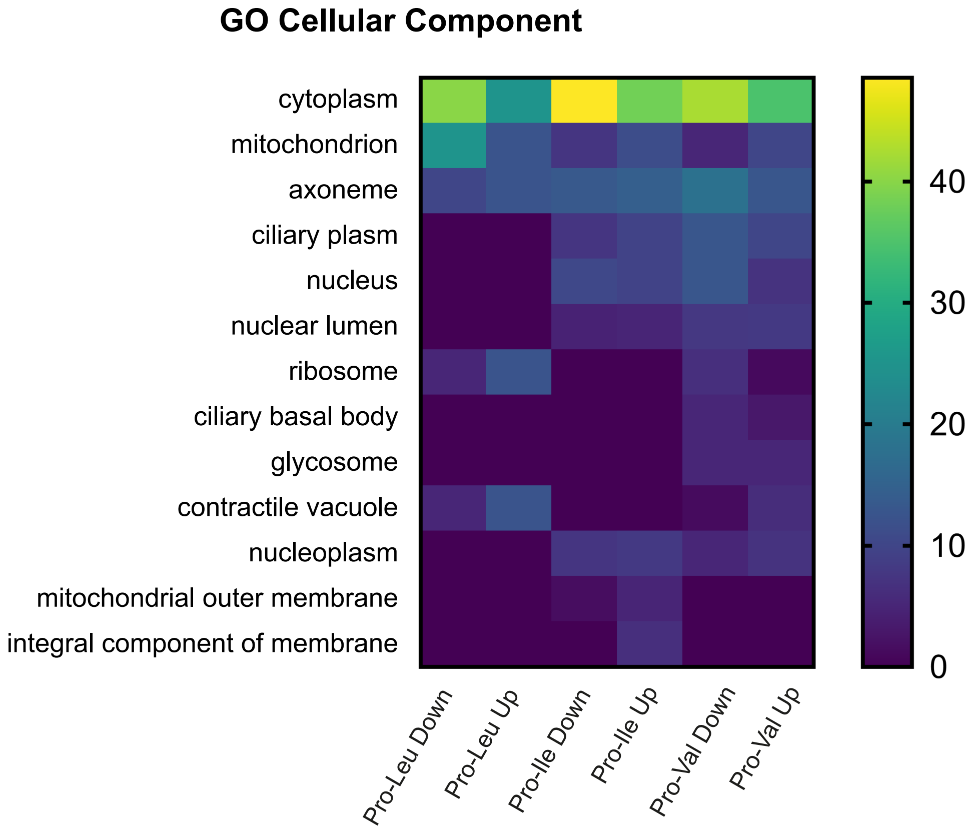

Figure S3: Heatmaps of A. GO Biological Process and B. GO Cellular Component. The frequency of each GO was normalized over the number of differentially expressed proteins in each group (Pro-Leu, Pro-Ile and Pro-Val).
