# Supplementary material for: Branched-chain amino acids modulate the proteomic profile of Trypanosoma cruzi metacyclogenesis induced by proline
Source: PLoS Negl Trop Dis. 2024 Oct 9;18(10):e0012588. doi: 10.1371/journal.pntd.0012588 (PMC11493278; doi:10.1371/journal.pntd.0012588)
Supplement: S4 Fig — A. Representative image of western blot for tyrosine aminotransferase (TAT) (~47 KDa) with total protein loading control stained with trichloroethanol (TCE) (left) and relative expression of TAT in the total extract of two biological replicates of metacyclics parasites (right). B. Average LFQ values for the four annotated TATs in metacyclics differentated in TAU Pro and TAU Pro-BCAAs. Graphs show average and standard deviation of three biological replicates. Statistically analysis using TAU Pro as the control was performed applying one-way ANOVA with multiple comparisons test (a = 0.05, ** p ≤ 0.01, *** p ≤ 0.001). (PDF) [file pntd.0012588.s004.pdf]

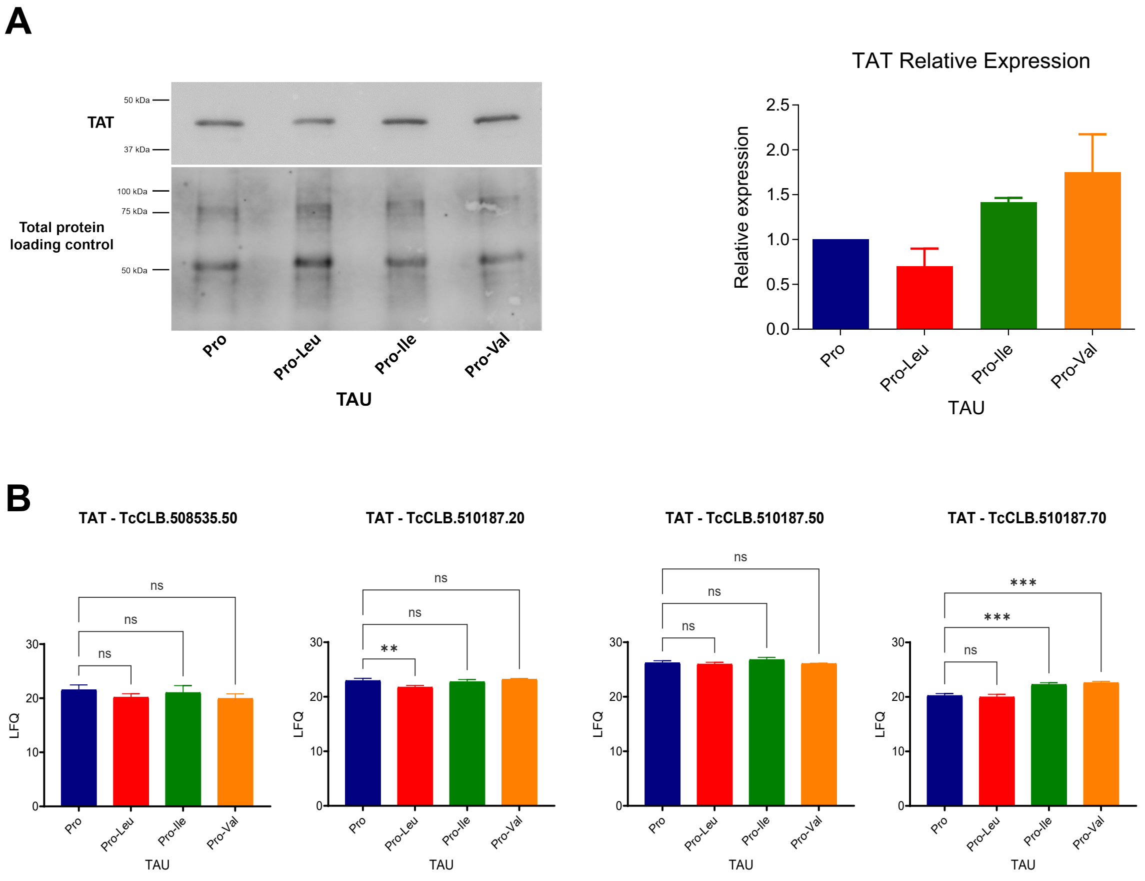

Figure S4: Biological validation of TAT protein levels in total extract of metacyclics differentiated in the presence of TAU Pro-BCAAs. A. Representative image of western blot for tyrosine aminotransferase (TAT) (~47 KDa) with total protein loading control stained with trichloroethanol (TCE) (left) and relative expression of TAT in the total extract of two biological replicates of metacyclics parasites (right). **B.** Average LFQ values for the four annotated TATs in metacyclics differentated in TAU Pro and TAU Pro-BCAAs. Graphs show average and standard deviation of three biological replicates. Statistically analysis using TAU Pro as the control was performed applying one-way ANOVA with multiple comparisons test (a=0.05, ** $p \leq 0.01$, *** $p \leq 0.001$).
