# Supplementary material for: Branched-chain amino acids modulate the proteomic profile of Trypanosoma cruzi metacyclogenesis induced by proline
Source: PLoS Negl Trop Dis. 2024 Oct 9;18(10):e0012588. doi: 10.1371/journal.pntd.0012588 (PMC11493278; doi:10.1371/journal.pntd.0012588)
Supplement: S5 Fig — A. Pyruvate kinase (PK) activity in total protein extracts of metacyclics differentated in TAU Pro and TAU Pro-BCAAs. Graphs show average and standard deviation of two biological replicates, with two technical replicates each. Statistically analysis using TAU Pro as the control was performed applying Turkey’s multiple comparisons test (a = 0.05, * p ≤ 0.05). B. Average LFQ values for the two annotated PK paralogues in metacyclics differentated in TAU Pro and TAU Pro-BCAAs. Graphs show average and standard deviation of three biological replicates. Statistically analysis using TAU Pro as the control was performed applying one-way ANOVA with multiple comparisons test (a = 0.05, ** p ≤ 0.01, *** p ≤ 0.001). (PDF) [file pntd.0012588.s005.pdf]

A

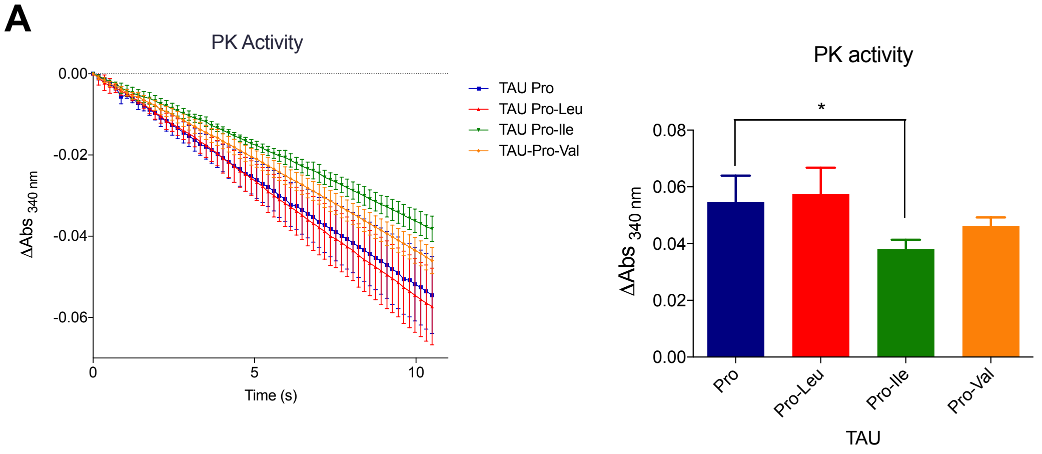

B

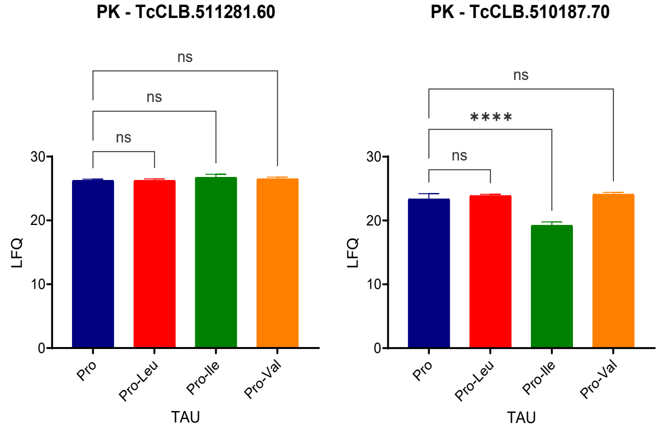

Figure S5: Biological validation of PK protein levels in total extract of metacyclics differentiated in the presence of TAU Pro-BCAAs. A. Pyruvate kinase (PK) activity in total protein extracts of metacyclics differentated in TAU Pro and TAU Pro-BCAAs. Graphs show average and standard deviation of two biological replicates, with two technical replicates each. Statistically analysis using TAU Pro as the control was performed applying Turkey's multiple comparisons test (a=0.05, * $p \leq 0.05$). **B.** Average LFQ values for the two annotated PK paralogues in metacyclics differentated in TAU Pro and TAU Pro-BCAAs. Graphs show average and standard deviation of three biological replicates. Statistically analysis using TAU Pro as the control was performed applying one-way ANOVA with multiple comparisons test (a=0.05, ** $p \leq 0.01$, *** $p \leq 0.001$).
