# Supplementary material for: Branched-chain amino acids modulate the proteomic profile of Trypanosoma cruzi metacyclogenesis induced by proline
Source: PLoS Negl Trop Dis. 2024 Oct 9;18(10):e0012588. doi: 10.1371/journal.pntd.0012588 (PMC11493278; doi:10.1371/journal.pntd.0012588)
Supplement: Attachment — Submitted filename: Response to reviewers.docx [file pntd.0012588.s033.docx]

**Responses to Reviewers’ Questions**

**About the methods:**

**Reviewer #1:** Methods all fine.

**Reviewer #2:** The experimental workflow is adequate, the data analysis robust and comparisons revealed a proteome remodelling snapshot where valine emerged as the most influential treatment.  Overall protein coverage was 7.6% (1,457 out of 19,242 proteins for CL-Brener proteome).  Changes in protein abundances depict a phenotype mainly driven by metabolic responses, attenuated translation and proteolytic rates, and changes in flagellar and parasite cell surface components.  While the study didn't pinpoint exactly which proteins control the parasites' transformation, it still provides valuable insights. On a technical note, trypanosomes proteome is poorly annotated, and this type of studies may improve the limited annotation present in nearly 33% of hypothetical proteins.

**Answer:**

We are acknowledged to the Reviewers for the positive comments.

--------------------

**About the results:**

**Reviewer #1:** In part. As detailed in my comments ot authors, the basic analysis of fine, it it the higher level interpretation that is problematic.

**Reviewer #2:** (No Response)

**Answer:**

Reviewer #1 please refer to our specific comments below.

--------------------

**About the conclusions:**

**Reviewer #1:** The conclusions are not fully realised or connected with the data.

**Reviewer #2:** (No Response)

**Answer:**

Reviewer #1 please refer to our specific comments below.

--------------------

**About the Editorial and Data Presentation Modifications:**

Reviewer #1: (No Response)

**Reviewer #2:** The data presented warrants publication in PNTD and I have made a few suggestions that can aid readers to better grasp the most relevant outputs of this study.

Major points:
-       The proteomic data provide evidence of a potential role for TAT as a frontline sensing component when free amino acids are the main carbon and nitrogen sources found in the culture media.  It will be informative to presenting an average LFQ comparison across the media conditions assayed for TAT and other metabolic components such as PK which stood out as proteins whose abundance was remarkably changed. Bars plot would be enough.

**Answer:**

We acknowledge the reviewer for the comment. We modified the manuscript to include the suggested changes. Fig. S4 was split into S4 and S5, where S4 contains the requested data (LFQ) for TAT and S5 for PK. For TAT we considered the 4 annotated paralogs and for PK the 2 annotated paralogs.

**Reviewer #2:**

-       The FAZ4, Gb4 and autophagy-related protein 24 are additional candidates that can be featured from this study in both abstract and the discussion sections. Is there any common feature in domain architecture or subcellular localisation among these? These protein hits might be worth studying further.

**Answer:**

The authors agree that the differentially regulated proteins, in common in all three conditions (nine in total), are very interesting candidates as regulators of metacyclogenesis. We added a statement indicating this in the abstract. Regarding FAZ4, Gb4 and ATG24, all of them are discussed in the discussion section. We have previously looked at all of them in detail and did not find any common features. Based on the information available on the TritrypDB and in the literature, these proteins have different locations, protein domains/structures and, very likely, function in unrelated biological processes. For that reason, we did not add any comment on this matter in the manuscript.

Here is a summarized list of features for each protein:

**Down-regulated proteins:**

TcCLB.503959.78 – Hypothetical protein – exclusively present in *T. cruzi* (among the trypanosomatids), contains a DUF1479 domain and structural prediction matches the ybiU protein from *E. coli*. Transcriptome data shows higher expression in epimastigotes. Unknown subcellular localization.

TcCLB.504203.40 – Hypothetical protein – mitochondrial protein with unknown function. Transcriptome data shows higher expression in epimastigotes.

TcCLB.506147.110 – Flagellum attachment zone protein 4 – protein localized in the FAZ, expressed in epimastigotes, metacyclics, and trypomastigotes. Phosphorylated during metacyclogenesis. Can be sumoylated. Ortholog in *Trypanosoma brucei* has mRNA expression regulated by RBP10.

TcCLB.510055.50 – Hypothetical protein - Transcriptome data shows low expression in all life stages, but more expressed in epimastigotes. Contains a forkhead-associated (FHA) domain. It has been found to be associated with chromatin.

TcCLB.510687.120 – snoRNP protein GAR1 – In *T. brucei* is localized in the nucleus and binds RNAs. Putatively involved in pseudouridine synthesis and ribosome biogenesis. Transcriptome data shows higher expression in epimastigotes.

**Up-regulated proteins:**

TcCLB.506177.20 – Lectin – Protein of unknown localization and function, similar to vesicular integral-membrane protein VIP36

TcCLB.508507.40 - Hypothetical protein - Transcriptome data shows low expression in all life stages, but is down-regulated in metacyclics. It has been found in the cell surface proteome. Can be sumoylated.

TcCLB.510101.450 - Hypothetical protein - Transcriptome data shows higher expression in metacyclics. Similar to a hypothetical protein present in Bodo saltans.

TcCLB.510749.30 - Autophagy-related protein 24 - Transcriptome data shows higher expression in metacyclics. RNAi in T. brucei causes enlargement of the flagellar pocket and induces cell differentiation. In T. brucei association of ATG24 to endocytic vesicles requires VPS34.

**Reviewer #2:**

-       It is fascinating to see that a single isomeric modification (i.e., leucine x isoleucine) can be decoded by the parasite and finely influence proteome changes. Another highlight.

**Answer:**

We are thankful to the Reviewer for the comment.

**Reviewer #2:**

Minor points:
-       The use of the acronym ‘pro’ is excessive and might add some confusion as this can also be interpreted as synonym of enhancer/promoter/inducer. I suggest naming it on its full extension throughout the main sections (tittle, abstract, conclusions).  For example, sentence in line 106: ‘Given that i) Pro is a pro-metacyclogenic amino acid’ can be misleading.

**Answer:**

The authors agree with the reviewer. The acronym Pro, together with other amino acids acronyms, has been replaced for its full name where appropriate.

**Reviewer #2:**

-       Using the term ‘protein expression’ is not adequate for the context of this type of study. Although it’s been widely used the word expression should only refer to gene expression. In the context of gene-encoded proteins I would suggest replacing expression for protein synthesis, protein levels or protein abundance. Protein expression is conceptually flawed.

**Answer:**

The authors thank the reviewer for the suggestion. We carefully reviewed the manuscript and replaced ‘protein expression’ and similar terms for either ‘protein levels’ or ‘protein abundance’. As we did not assess protein synthesis in this study and variations in protein levels could be due to other processes (such as reduced/increased protein turnover) we refrained from using ‘protein synthesis’ as a synonym of protein levels.

**Reviewer #2:**

-       Line 340: How do the authors infer metabolic changes from proteome data? The variations observed in enzymes of glutamate, proline, serine, threonine and glutamine metabolism reflect an overall metabolic remodelling that, in my view, is mostly driven by the metabolic condition imparted in the cultivation media.

**Answer:**

The authors apologize for the mistake, where it reads ‘metabolic changes’ it should read ‘proteome changes’. It has been corrected in the manuscript.

**Reviewer #2:**

-       Comparisons between protein changes observed in TAU-proline x TAU-proline+valine (Lines 360-70) are not properly presented as the changes highlighted do not specify in what condition the up/down regulated hits are enriched. For example, in which comparison were the 78 downregulated hits seen? Similarly written in line 376. Please note that data presented in lines 389-409 are much easier to follow on and correlate with the supporting figures.
**Answer:**

The authors agree with the reviewer. To clarify this point, we added the description of the comparisons that were performed to find the up and down-regulated proteins to lines 349-350 and 369-370 (line numbers refer to the version with track changes).

**Reviewer #2:**

-       Line 459-60 (Discussion section) where the authors state the difference in substrate preferences might reflect specific nutritional needs throughout life cycle. I think it would be useful to elaborate more around this claim and, if possible, provide a documented example.

**Answer:**

Indeed, this part of the discussion is speculative, as the transport and intracellular levels of BCAAs have not been characterized in the different life stages of *T. cruzi*. However, metabolic preferences by different stages and even in different proliferation phases of one stage, are well-established facts in *T. cruzi*. One documented example of a specific nutritional need is the requirement for proline and glucose in the different life stages of *T. cruzi*. In the tissue-derived trypomastigotes, both glucose transport and its transporter reach maximum values while proline transport activity is at its minimum value when compared to other stages. In the intracellular epimastigote stage, however, the proline transport is the highest when compared to other life forms (Silber et al, 2009). Accordingly, expression of the first enzyme of the proline oxidation pathway, ProDH, is up-regulated in this life stage (Paes et al, 2013) showing a metabolic preference for this amino acid in this life stage.

**Reviewer #2:**

-       Do the authors think that an increased amino acid catabolic activity can be a trigger of metacyclogenesis arrest? This is put in the context of increased GS activity in the TAU-proline plus valine dependent metacyclogenesis.

**Answer:**

Several amino acids have been reported to support metacyclogenesis as the sole carbon and energy source such as proline, glutamine and histidine (Contreras et al, 1985; Homsy et al, 1989, Damasceno et al, 2018, and our unpublished data). BCAAs alone do not support metacyclogenesis and have been shown to reduce metacyclogenesis induced by proline (Rapado et al, 2023 and this work). However, the mechanisms by which BCAAs inhibit metacyclogenesis have not been elucidated. It is worth clarifying that in this work we purified the metacyclics differentiated in the presence of Pro alone or combined with each BCAA. Therefore, we only analysed cells which likely “escaped” the inhibition of the metacyclogenesis caused by the BCAAs. Being TAT responsible for the first step of the BCAAs degradation pathway, the transamination, (Manchola et al, 2018) and GS an ammonium detoxifier in *T. cruzi* (Crispim et al, 2018) it is reasonable to speculate that parasites that up-regulate both enzymes can different despite the presence of BCAAs.

--------------------

**About the** Summary and General Comments

**Reviewer #1:**

This is an interesting paper that seeks to address the question of the impact of different amino acids on differentiation in T. cruzi. Given that differentiation is frequently associated with stress conditions, understanding the metabolic consequences required for and arising from differentiation is important to understand the basic biology behind the process. Technically I have no real criticisms or comments as the proteomics approach is absolutely standard and clearly carried out correctly. There is some noise revealed in the PCA analysis in F1 revealing an outlier for several of the conditions, but that is unlikely to strongly impact the data.

**Answer:**

We are acknowledged to the Reviewer for the comment.

**Reviewer #1:**

My real issue has to do with the manner in which the data are considered. As we work through the results there is little interpretation, but when we come to the discussion I was struck that the vast majority of this could have been written without the proteomics having been done. There is little reference back to this except for the section on the flagellar changes. I also worry that considerable issues arise from the use of GO terms which are poor for kinetoplastids and a lack of detailed analysis of more prominent changes. This seems to be a missed opportunity and I would suggest improving the discussion; the authors are well respected in this area and their insights would be valuable.

**Answer:**

We are deeply acknowledged to the Reviewer for his/her consideration. A concern in writing this kind of manuscript is to avoid being repetitive about the results, which are vastly exposed in the corresponding section. Thus, we used a common structure in most of the paragraphs in the discussion section: they begin with a description of the process that will be discussed, followed by a brief description/comment on the data we found, and end with a contextualization of what was published previously. For more detail, our results are referenced in the discussion in the following lines (line numbers refer to the version with track changes):

Lines 486 – 493 – TAT and GS up-regulation

Lines 499 – 502; 506 – 512 – antioxidant defence

Lines 516 -523 – translation

Lines 528 – 535 – flagellar structure

Lines 575 – 577 – trans-sialidases

Lines 584 – 586 – autophagy

**Reviewer #1:**

Some specific things are below;

Suggest Pro-induced is a bit cryptic in the title.

**Answer:**

The authors agree with the reviewer. We replaced ‘Pro-induced’ with ‘induced by proline’ in the title and throughout the manuscript.

**Reviewer #1:**

Grammer odd for first sentence of abstract.

**Answer:**

The authors agree and have rephrased the sentence.

**Reviewer #1:**

down-regulated proteins are involved in biological processes such as the generation of energy, posttranscriptional regulation of gene expression and oxidation-reduction processes; on the other hand, up-regulated proteins participate in respiration and translation processes
Not sure I quite grasp the distinction here - respiration is an energy generator and posttranscriptional events include translation.

**Answer:**

We understand the Reviewer´s point. This problem is derived from the fact that we decided to stick to the broadly used GO categories, which in some cases are somehow conflicting. One explanation for this is that the descriptors do not necessarily have the same category level, which brings us to the pointed overlaps and contradictions. For example, the generation of energy is a wider category than respiration, which is a particular case of it. The same applies to ‘post-transcriptional events’ which is more general than and includes translation.

**Reviewer #1:**

Define TAT in discussion.

**Answer:**

Done as requested.

**Reviewer #1:**

The differences in the metabolic fates of the BCAAs might explain the phenotypic particularities observed in the metacyclic trypomastigotes differentiated in the presence of these amino acids.
Yes, but how?

**Answer:**
Currently, our lab is trying to decipher exactly how each BCAA produces the various characteristics that are being observed, but we do not have a final answer. Experimental data from our group and others demonstrate that the parasite when maintained/cultured under conditions that allow the utilization of each BCAA independently displays different phenotypes (Mancilla, Naquira & Lanas 1966; Homsy et al, 1989; Ginger, Prescott, Reynolds et al, 2000; Manchola et al, 2018; Rapado et al, 2023; Souza ROO et al, in preparation; Marsiccobetre et al, in preparation, both from our research group). We modified some of the discussion to exemplify the phenotypical characteristics of these parasites, but we do not have an explanation of the mechanisms yet. Of course, this is being a major matter of research in our laboratory.

**Reviewer #1:**

In our case, some specific putative trans-sialidases proteins are downregulated in trypomastigotes obtained in TAU Pro-Leu and TAU Pro-Val when compared to metacyclics obtained in TAU Pro. How does the present dataset compare to the other studies cited?

**Answer:**

As previously mentioned in the discussion, it has been shown that during metacyclogenesis all trans-sialidases are up-regulated (De Godoy et al, 2012). The fact that differentiation in the presence of Pro-Leu and Pro-Val causes down-regulation of some trans-sialidases might help to explain the phenotype previously observed by our group that metacyclics differentiated in the presence of Pro+BCAAs are less infective (Rapado et al, 2023). Additionally, it has been recently shown that knocking out active trans-sialidases impairs the intracellular differentiation from amastigote to trypomastigote (Burle-Caldas et al, 2022). These pieces of information have now been added to the manuscript.

**Reviewer #1:**

Odd that if autophagy is activated that only see ATG24 and none of the catabolic or trafficking enzymes are noted.

**Answer:**

Indeed, the authors carefully reviewed the dataset and did not find any of the other proteins putatively involved in autophagy. It could be due to a limitation of our dataset, as we detected only 1457 proteins common in all analysed samples.

**Reviewer #2:**

Professor Silber’s research group has greatly increased our understanding of how amino acids influence the trypanosomatids’ life cycle. Multiple lines of evidence have demonstrated that its functionality goes beyond its proteogenic role playing a pivotal role in parasite proliferation/infectivity, energy production, osmoregulation, and stress response. Particularly, proline acts as modulator of parasite differentiation and mitochondrial homeostasis.

In this study the authors applied bottom-up proteomic profiling to delve into the function of branched chain amino acids (leucine, isoleucine and valine) during the differentiation process known as metacyclogenesis (i.e., cellular transformation from epimastigote to metacyclic trypomastigote).  For this characterisation authors applied a well-established system of axenic cultivation in nutrient-scarce media, followed by affinity-based cell purification, which enabled the assessment of proteome-wide changes upon proline-dependent metacyclogenesis.

**Answer:**

We are deeply acknowledged to the Reviewers for their constructive and detailed review of our work. No doubt their work gave us the chance to improve it.
